# Short-Term Outcomes of Patients With Hyponatremia Presenting to the Emergency Department: An Observational Study

**DOI:** 10.7759/cureus.63679

**Published:** 2024-07-02

**Authors:** Rakesh G Shekar, Mahaveer Singh Rodha, Ankur Sharma, Amit Rohila, Kamla Kant Shukla, Rahul Choudhary, Gopal K Bohra

**Affiliations:** 1 Trauma and Emergency, All India Institute of Medical Sciences, Jodhpur, Jodhpur, IND; 2 Trauma and Emergency/General Surgery, All India Institute of Medical Sciences, Jodhpur, Jodhpur, IND; 3 Trauma and Emergency/Anesthesia and Critical Care, All India Institute of Medical Sciences, Jodhpur, Jodhpur, IND; 4 Emergency Medicine, All India Institute of Medical Sciences, Jodhpur, Jodhpur, IND; 5 Biochemistry, All India Institute of Medical Sciences, Jodhpur, Jodhpur, IND; 6 Cardiology, All India Institute of Medical Sciences, Jodhpur, Jodhpur, IND; 7 Medicine, All India Institute of Medical Sciences, Jodhpur, Jodhpur, IND

**Keywords:** mortality, icu, sodium, emergency, hyponatremia

## Abstract

Background: Hyponatremia is the predominant electrolyte imbalance disorder in the emergency department. It can manifest with a diverse array of symptoms, ranging from non-specific and moderate to severe and even life-threatening. There is a scarcity of literature addressing the clinical characteristics and prognosis of patients with hyponatremia presenting to the emergency department in the western part of Rajasthan. The objective of this study was to investigate the impact of hyponatremia on the outcomes of patients presenting to the emergency department.

Methods: In this prospective, cross-sectional, observational study, 200 patients aged more than 18 years who presented to the emergency department with serum sodium < 135 mEq/l were included. The triage of patients was determined by their primary complaints. The primary outcome was to study the clinical profile of patients with hyponatremia presenting to the emergency department. The secondary outcomes were to examine the etiology, i.e., hypovolemic, euvolemic, or hypervolemic, and the outcome of patients on the 7th day (patient admitted to the ward or intensive care unit) and the 28th day (discharged or death) with hyponatremia presenting to the emergency department. The clinical status of the patients was noted by telephonic follow-up in case they were not admitted for this period.

Results: Out of 200 patients, 66 (33%) had hypovolemic, 96 (48%) had euvolemic, and 38 (19%) had hypervolemic hyponatremia. We observed that seizures (84.2%), confusion (56%), and coma (77.7%) were the most common clinical features of patients with severe hyponatremia in the emergency, which was statistically significant than mild and moderate hyponatremia (p = 0.03, 0.023, and 0.029, respectively). On the 7th day of hospitalization, out of 181 (90.5%) admissions in the ward, 116 (64.08%) had severe hyponatremia, and out of 19 (9.5%) ICU admissions, 13 (68.4%) had severe hyponatremia. Death was seen in five (2.5%) patients, one (20%) in moderate and four (80%) in severe hyponatremia cases.

Conclusion: Most cases of hyponatremia in this study were euvolemic. Most patients experienced severe hyponatremia, and seizures, confusion, and coma were the most prevalent symptoms. These disorders must be recognized early to properly diagnose and treat hyponatremia and prevent its morbidity and death.

## Introduction

Hyponatremia is the most commonly observed disorder of fluid and electrolyte balance in clinical practice [1]. It is present in 15-20% of hospital emergency admissions and up to 20% of patients who are critically ill [2,3].

Hyponatremia can cause a wide range of symptoms, from non-specific and mild, such as weakness, spasms, cramps, fatigue, nausea, irritability, memory loss, and headaches, to severe and potentially fatal, such as seizures, decreased consciousness, coma, and cardiorespiratory distress [4,5]. Acute severe hyponatremia can cause lifelong brain injury, respiratory arrest, brain herniation, and death. Hyponatremia symptoms vary according to their onset, duration, and severity [6]. Symptoms are more prevalent in individuals with acute hyponatremia than in those with chronic hyponatremia due to brain volume adaptation, a mechanism that corrects hypotonicity-induced brain swelling [7]. The various clinical presentations, underlying causes, and potential for catastrophic consequences of this condition contribute to the complexity of patient care. It frequently indicates an imbalance in the regulation of bodily fluids, which can result from different underlying illnesses such as congestive heart failure, liver cirrhosis, and renal disease [6]. The difficulty of diagnosis, presence of concomitant illnesses, requirement for rigorous monitoring and therapy, risk of consequences, unpredictability in treatment response, and management of underlying causes can all contribute to lengthier hospital hospitalizations. Hyponatremia contributes to higher healthcare expenses due to prolonged hospital stays, extensive surveillance, treatment, and adverse outcomes [7].

There are limited studies in the literature regarding the clinical profile and outcome of patients with hyponatremia presenting to the emergency department of this part of the world, i.e., in the western part of Rajasthan, India, especially Jodhpur. It will help to determine the best use of available resources for hyponatremia management. Therefore, we planned this study to see whether the presence of hyponatremia affects the outcome of patients received in the emergency department.

## Materials and methods

This prospective, cross-sectional, observational study was carried out in the emergency department of a tertiary care hospital between September 2021 and June 2023. After obtaining ethical clearance from the institute's ethical committee (AIIMS/IEC/2021/3601), all the patients of age >18 years presenting to the emergency department with serum sodium < 135 mEq/l were included in the study. If a blood report was unavailable during the emergency presentation, arterial or venous blood gas was used to diagnose patients with hyponatremia. Patients of hyperglycemia with random blood sugar levels > 200 mg/dl, chronic kidney disease on maintenance hemodialysis, hyperlipidemia with total cholesterol levels > 200 mg/dl, and multiple myeloma were excluded, as these conditions can decrease the sodium level in the blood. Informed written consent was obtained from all the participants. If the patient was in altered sensorium, then consent was taken from the family member.

Patients coming to the emergency were triaged based on their chief complaints. After inclusion in the study, blood and urine samples were collected in a sterile container.

Plasma osmolality was calculated based on the following formula: 2 x [Na+] (meq/L) + glucose (mg/dl)/18 + blood urea nitrogen (mg/dl)/2.8. The clinical profile of patients with hyponatremia was studied. Based on the clinical findings and lab reports, hyponatremia was classified based on the etiology. Based on the lab reports, hyponatremia was classified as mild (130-134 mEq/L), moderate (125-129 mEq/L), and severe (<125 mEq/L). It was further classified based on clinical findings and volume status as hypovolemic, euvolemic, and hypervolemic hyponatremia. Accordingly, the patients were managed in the emergency, and their outcome was assessed on day seven and day 28 personally or telephonically. The primary objective was to study the clinical profile (demographic parameters, comorbidities, symptoms, drugs received, imaging, correction given, etc.) of patients with hyponatremia presenting to the emergency department. The secondary objectives were to study the etiology (hypovolemic, euvolemic, or hypervolemic) and outcome of patients on the 7th day (patient admitted in the ward or intensive care unit) and the 28th day (discharged or death) with hyponatremia presenting to the emergency department. The clinical status of the patients was noted by telephonic follow-up in case they were not admitted for this period.

Cumming et al. reported the prevalence of hyponatremia in their study as 13.4% [8]. Using this for sample size calculation, we estimated a sample size of 200 patients considering a 10% dropout rate using the formula (n = (z)2 p ( 1 - p ) / d2), where n is the sample size, z is the statistic corresponding to 95% level of confidence (1.96), p is prevalence (13.4), and d is tolerated margin of error (5%).

Statistical analysis

SPSS version 21.0 statistical program (IBM Corp., Armonk, NY) was used to analyze the data. To describe nominal data, frequency and percentages were utilized, and the chi-squared test was used to compare these data. Continuous data were expressed as mean ± standard deviation and compared using an unpaired t-test. P-values less than 0.05 were deemed statistically significant.

The reporting for this observational study complies with the STROBE (Strengthening the Reporting of Observational Studies in Epidemiology) recommendations.

## Results

**Figure 1:**
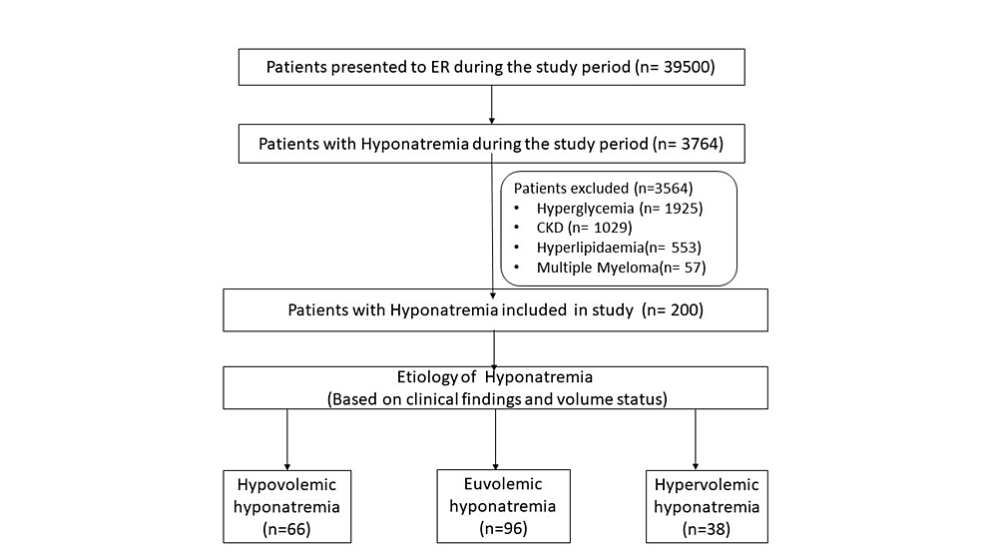
Flow diagram of participants in the study. ER: emergency room; CKD: chronic kidney disease.

During the study period, 3794 patients had hyponatremia due to various reasons. Out of these, 3564 patients were excluded for multiple reasons. Finally, 200 patients with hyponatremia were included in this study, as shown in Figure 1.

Clinical profile, symptoms, comorbidities, drugs received, imaging, and correction given for hyponatremia to patients are shown in Table 1.

**Table 1:** Baseline characteristics of patients presenting to the emergency department. M: male; F: female; GCS: Glasgow Coma Scale; CT: computed tomography; NS: normal saline.

Parameter				Total patients (n = 200)	Mild hyponatremia	Moderate hyponatremia	Severe hyponatremia	P-value
Patient information								
Age	<60 years			75	7 (9.3%)	26 (34.66%)	42 (56%)	0.146
	>60 years			125	9 (7.2%)	29 (23.2%)	87 (69.6%)	
Sex	M			95	7 (7.3%)	26 (27.3%)	62 (65.2%)	0.974
	F			105	9 (8.5%)	29 (27.6%)	67 (63.8%)	
Clinical presentation								
Weakness				83	7 (8.4%)	24 (28.9%)	52 (62.6%)	0.873
Vomiting				62	3 (4.3%)	21 (33.8%)	38 (61.2%)	0.316
Seizure				19	0 (0%)	3 (15.2%)	16 (84.2%)	0.03
Confusion				78	3 (3.8%)	19 (24.3%)	56 (71.7%)	0.023
Coma				7	0 (0%)	2 (28.57%)	5 (71.42%)	0.029
GCS		13-15		129	13 (10.07%)	39 (30.2%)	77 (59.6%)	0.399
		8-12		64	3 (4.6%)	14 (21.8%)	47 (73.4%)	
		<8		7	0 (0%)	2 (28.57%)	5 (71.42%)	
Past medical history								
Cardiac failure				11	1 (9%)	3 (27.2%)	7 (63.6%)	1.00
Renal failure				10	2 (20%)	1 (10%)	7 (70%)	0.181
Liver cirrhosis				12	0 (0%)	5 (41.6%)	7 (58.3%)	0.493
Stroke				19	1 (5.2%)	3 (15.7%)	15 (78.9%)	0.507
Hypertension				69	6 (8.6%)	16 (23.1%)	47 (68.1%)	0.593
Hypothyroid				22	3 (13.6%)	10 (45.4%)	9 (40.9%)	0.36
On treatment								
Receiving drug			Diuretic	23	1 (4.3%)	6 (26.08%)	16 (69.5%)	0.949
			Other drug	10	1 (10%)	2 (20%)	7 (70%)	
CT scan of the brain								
Normal				73	2 (2.7%)	19 (26.02%)	52 (71.2%)	0.138
Abnormal				28	2 (7.1%)	6 (21.4%)	20 (71.4%)	
Hyponatremia correction by								
0.9% NS				85	10 (11.7%)	33 (38.8%)	42 (49.4%)	0.037
3% NS				75	0 (0%)	15 (20%)	60 (80%)	
Diuretics				35	5 (14.2%)	7 (20%)	23 (65.7%)	

Seizures (84.2%), confusion (71.7%), and coma (71.42%) were the most common clinical feature of patients with severe hyponatremia in the emergency, which was statistically significant than mild and moderate hyponatremia (p = 0.03, 0.023, and 0.029, respectively). Most of the patients were receiving diuretics. Other drugs received by the patients were escitalopram (two patients), sodium valproate (four patients), oxcarbamazepine (three patients), and quetiapine (one patient).

**Figure 2:**
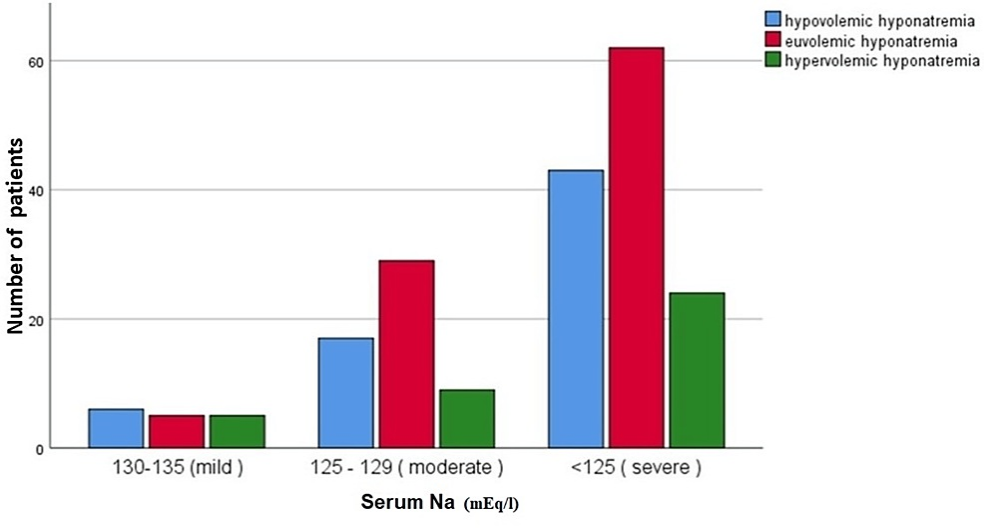
Bar chart showing the etiology of hyponatremia of patients presenting to the emergency department. Na: sodium.

In our study, 16 (8%) patients had mild hyponatremia, out of which six (37.5%) were hypovolemic, five (31.25%) were euvolemic, and five (31.25%) were hypervolemic. Fifty-five (27.5%) patients had moderate hyponatremia, out of which 17 (30.9%) were hypovolemic, 29 (52.7%) were euvolemic, and nine (16.36%) were hypervolemic. A total of 129 (64.5%) patients had severe hyponatremia, out of which 43 (33.33%) were hypovolemic, 62 (48.06%) were euvolemic, and 24 (18.6%) were hypervolemic (Table 2 and Figure 2).

**Table 2:** Etiology of hyponatremia of patients presenting to the emergency department.

Etiology	Mild	Moderate	Severe	P-value
Hypovolemic hyponatremia	6 (37.5%)	17 (30.9%)	43 (33.33%)	0.585
Euvolemic hyponatremia	5 (31.25%)	29 (52.7%)	62 (48.06%)	
Hypervolemic hyponatremia	5 (31.25%)	9 (16.36%)	24 (18.6%)	

There was also a statistically significant difference in urine sodium values in various hyponatremia etiologies (Table 3).

**Table 3:** Urine sodium in various etiologies of hyponatremia.

Etiology	Urine sodium (Ur Na)		P-value
	<20 meq/L	>20 meq/L	
Hypovolemic hyponatremia	23 (52.57%)	43 (27.5%)	0.01
Euvolemic hyponatremia	10 (22.7%)	86 (55.12%)	
Hypervolemic hyponatremia	11 (25%)	27 (17.3%)	

In our study, 116 patients admitted with severe hyponatremia were treated in the ward. They were not on any support and could be managed in the ward with only hyponatremia correction.

**Figure 3:**
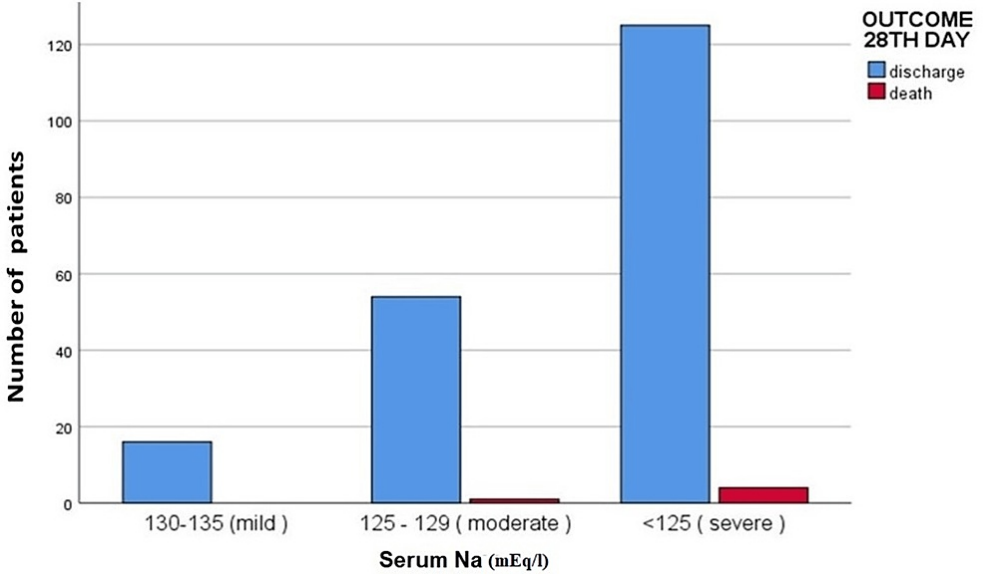
Bar chart showing outcome on the 28th day in patients with hyponatremia presenting to the emergency department. Na: sodium.

On the 28th day of follow-up, 195 (97.5%) patients were discharged, which included 16 (8.2%) mild, 54 (27.69%) moderate, and 125 (64.1%) severe hyponatremia patients. Death was seen in five (2.5%) patients, one (20%) in moderate and four (80%) in severe hyponatremia cases (Table 4 and Figure 3).

**Table 4:** Outcome of patients with hyponatremia presenting to the emergency department.

		Total patients (n = 200)	Mild hyponatremia	Moderate hyponatremia	Severe hyponatremia	P-value
7^th^ day	Patients in ward	181	15 (8.2%)	50 (27.6%)	116 (64.08%)	1.00
	Patients in ICU	19	1 (5.2%)	5 (26.3%)	13 (68.4%)	
28^th^ day	Discharge	195	16 (8.2%)	54 (27.69%)	125 (64.1%)	1.00
	Death	5	0 (0%)	1 (20%)	4 (80%)	

## Discussion

Hyponatremia is frequently observed in emergency rooms and has been found to increase mortality, mainly when it is severe [9]. In the current study, the most prevalent cause was euvolemic hyponatremia. It necessitated admission to the ICU for 9.5% of patients, while the majority (90.5%) were admitted to wards. To accurately diagnose and treat hyponatremia and prevent morbidity and mortality, it is imperative to identify these disorders at an early stage.

Lee et al. found that the initial signs of patients who presented with hyponatremia were generalized weakness (33.3%), seizure (28.6%), altered mental status (21.6%), confusion (9.5%), dizziness (4.8%), and non-specific (2.4%) [10]. In Bokemeyer et al.'s study, of the 261 patients with hyponatremia, 140 exhibited neurological symptoms [11]. Most of these symptoms (59%) were general weakness and disorientation. Babaliche et al. observed vomiting and confusion as the two most typical complaints in these patients [12]. In our study, the clinical presentation of patients with all grades (mild, moderate, and severe) of hyponatremia was weakness (41.5%), vomiting (31%), seizure (9.5%), confusion (39%), and coma (4.5%). In Mittal et al.'s study, when compared to mild hyponatremia, patients with severe hyponatremia had neurological symptoms more prevalent (69.7% versus 8.1%) [13]. Similarly, we observed that seizures (84.2%), confusion (56%), and coma (77.7%) were the most common clinical feature of patients with severe hyponatremia in an emergency, which was statistically significant than mild and moderate hyponatremia (p = 0.03, 0.023, and 0.029, respectively).

In Kaspa et al.'s study [14], comorbidities related to hyponatremia included cardiac issues (31%), renal issues (19%), respiratory issues (18%), hypertension (58%), and diabetes mellitus (55%). In our study, comorbidities related to hyponatremia included cardiac failure (5.5%), renal failure (5%), liver cirrhosis (6%), hypothyroidism (11%), hypertension (34.5%), and stroke (9.5%). We found that comorbidities were frequently seen in patients with severe hyponatremia compared to moderate and mild hyponatremia. We observed that 19 patients with prior stroke presented with altered sensorium due to hyponatremia in the present study.

Zhang et al. found that the medications most frequently linked to hyponatremia were proton pump inhibitors (PPIs) (59.7%), loop diuretics (57.4%), potassium-preserving diuretics (29.5%), angiotensin-converting enzyme (ACE) inhibitors/angiotensin receptor blockers (ARBs) (20.0%), thiazide diuretics (12.5%), and non-steroidal anti-inflammatory drugs (NSAIDs) (12.4%) [15]. In our study, diuretics (13 patients on loop diuretics and 10 on thiazide diuretics) were a cause of hyponatremia in 11.5% of patients, out of which 69.5% had severe hyponatremia. Other drugs, including escitalopram (two patients), sodium valproate (four patients), oxcarbamazepine (three patients), and quetiapine (one patient), causing hyponatremia were observed in 5% of patients.

In Prkačin et al.'s study, patients with hyponatremia in the emergency department were treated with 3% hypertonic saline [16]. In our study, out of the 85 patients who were managed with normal saline, 49.4% of patients had severe hyponatremia, and in 75 patients who were managed with 3% hypertonic saline, 80% of patients had severe hyponatremia. The patients usually presented to emergency with seizure, confusion, and coma and received a bolus of 3% hypertonic saline over 20-30 minutes. Diuretics were given in 23% of severe hyponatremia cases. These patients were primarily hypervolemic.

In Bokemeyer et al.'s study, focal neurological symptoms were present in 31% of cases, and neuroimaging was done on 68% of the symptomatic patients [11]. In our study, neuroimaging was done in 50.5% of patients, of which 36.5% had normal and 14% had abnormal neuroimaging. Most abnormal neuroimaging was associated with severe hyponatremia (71.4%), which included old infarcts, meningeal enhancement, cerebral edema, bleeding, and hydrocephalus.

Prkačin et al. found that improvement in patient clinical status was attained in 54% of patients with hyponatremia; 12% of patients did not survive the disease as a consequence of hyponatremia, whereas in 34% of patients, the clinical condition remained constant [10]. Isaak et al. found that patients who received treatment had considerably greater survival than those who did not [17]. More efforts should be made to ensure the treatment of severe hyponatremia in hospitalized patients. Severe hyponatremia resulted in a prolonged hospital stay. Additionally, moderate and severe hyponatremia were linked to a significant rise in in-hospital mortality, when compared to mild hyponatremia. In our study, most patients on the 7th day were admitted to wards (90.5%), while 9.5% of patients needed ICU admission, which was most commonly seen in severe hyponatremic patients.

Babaliche et al. [12] and Singh et al. [18] found that the most frequent cause of hyponatremia was euvolemic hyponatremia. In Mittal et al.'s study, 7.2% of patients had hypovolemic hyponatremia, 21.2% had hypervolemic hyponatremia, and 61.6% of patients had euvolemic hyponatremia [13]. In our study, 48.06% of patients with severe hyponatremia displayed euvolemia. Hypovolemia was seen in 33.33% and hypervolemia in 18.6% of severe hyponatremia. Thus, euvolemia was more common in our study.

This research has certain limitations. First and foremost, this is a single-center research. Our results may need to be more generalizable to other patient populations. In addition, the interpretation of variables causing hyponatremia was limited to one expert physician. Acute and chronic hyponatremia were not distinguished.

## Conclusions

Euvolemic hyponatremia was identified as the most common etiology in this study. Hyponatremia symptoms can range from vomiting, lethargy, and malaise to disorientation, seizures, and coma. In our study, the majority of patients (90.5%) with hyponatremia were admitted to wards, whereas 9.5% required ICU admission. Mortality was reported in 2.5% of individuals admitted to the ICU with multiple comorbidities. These disorders must be identified early to correctly diagnose and treat hyponatremia and avoid morbidity and mortality.
